# A clinical prediction model for outcome and therapy delivery in transplant-ineligible patients with myeloma (UK Myeloma Research Alliance Risk Profile): a development and validation study

**DOI:** 10.1016/S2352-3026(18)30220-5

**Published:** 2019-02-06

**Authors:** Gordon Cook, Kara-Louise Royle, Charlotte Pawlyn, Anna Hockaday, Vallari Shah, Martin F Kaiser, Sarah R Brown, Walter M Gregory, J Anthony Child, Faith E Davies, Gareth J Morgan, David A Cairns, Graham H Jackson

**Affiliations:** a Leeds Institute of Cancer and Pathology, University of Leeds, Leeds, UK; b Clinical Trials Research Unit, Leeds Institute of Clinical Trials Research, University of Leeds, Leeds, UK; c The Institute of Cancer Research, London, UK; d Myeloma Institute, University of Arkansas for Medical Sciences, Little Rock, AR, USA; e Department of Haematology, Newcastle University, Newcastle-upon-Tyne, UK

## Abstract

**Background:**

Tolerability of treatments for multiple myeloma can depend on the characteristics of the patient being treated. We aimed to develop and validate a risk profile, using routinely collected data, that could predict overall survival in patients with multiple myeloma who were ineligible for stem-cell transplantation.

**Methods:**

We used patient data from two randomised controlled trials done in patients with newly diagnosed multiple myeloma who were ineligible for stem-cell transplantation (the NCRI Myeloma XI study [NCRI-XI, n=1852] and the MRC Myeloma IX study [MRC-IX, n=520]), to develop the UK Myeloma Research Alliance Risk Profile (MRP) for overall survival. We used multivariable Cox regression with a least absolute shrinkage and selection operator penalty term. Multiple imputation by chained equations was used to account for missing data in the development and internal validation of the model. The MRP was internally validated in NCRI-XI and externally validated in MRC-IX. The D-statistic was estimated in the developed model and used to internally and externally validate the model according to prespecified criteria.

**Findings:**

The MRP included WHO performance status, International Staging System, age, and C-reactive protein concentration as prognostic variables. The MRP was prognostic of overall survival and was successfully internally validated in NCRI-XI and externally validated in MRC-IX (D-statistic NCRI-XI: 0·840 [95% CI 0·718–0·963] and MRC-IX: 0·654 [0·497–0·811]). The MRP groups defining low-risk, medium-risk, and high-risk patients were associated with progression-free survival and early mortality. A decrease in the percentage of protocol dose delivered and quality of life at baseline were associated with increased risk. The MRP groups remained prognostic in patients exposed to different therapeutic combinations and in patients with genetic high-risk disease defined according to both the UK and International Myeloma Working Group definitions.

**Interpretation:**

We have developed and externally validated a risk profile for overall survival containing widely available clinical parameters. This risk profile could aid decision making in patients with multiple myeloma ineligible for stem-cell transplantation, but further external validation is required.

**Funding:**

Medical Research Council, Novartis, Schering Health Care, Chugai, Pharmion, Celgene, Ortho Biotech, Cancer Research UK, Celgene, Merck Sharp & Dohme, and Amgen.

## Introduction

Multiple myeloma is molecularly heterogeneous, but the patients it affects are also heterogeneous. This heterogeneity becomes more notable in older, less fit patients in whom factors affecting their ability to withstand treatment might be more important than disease biology.^1^ Myeloma is predominantly a disease associated with advanced age, with 45% of patients aged 75 years and older at diagnosis;^2^ these patients represent a substantial proportion of newly diagnosed patients requiring treatment, yet they are historically underrepresented in clinical trials.^3^ Ageing is the leading risk factor for most chronic conditions that affect survival, independence, and wellbeing. These disorders, including atherosclerosis, cancer, dementias, and metabolic syndromes, are becoming progressively more prevalent as the elderly population increases. Age-related frailty syndrome is an entity defined as a heightened vulnerability to stresses (eg, surgery, infection, or trauma) coupled with sarcopenia and cachexia. Inflammation is the key physiological correlate of this syndrome, which predisposes to chronic disease, loss of independence, and mortality, and greatly increases health-care costs.^4^

Tolerability of anticancer therapy does not deteriorate linearly with age, so defining which patients are most at risk of outcome, measured with patient-reported or biophysical measurements, might enable better treatment stratification. The Katz Activity of Daily Living (ADL),^5^ Lawton's Instrumental Activity of Daily Living (IADL),^6^ and the Charlson Comorbidity Index (CCI)^7^ have been combined with age to derive the International Myeloma Working Group (IMWG) Frailty Score,^8^ which can predict patient outcome and has been prospectively externally validated in a single-centre study.^9^ Investigative work by several researchers has produced several variants of the frailty score devised by the IMWG.^8^, ^10^ However, the main limitations of these scoring systems are their inherent subjectivity and the time that they require to administer in the clinic. A simpler, objective tool is needed that defines subpopulations at risk of early mortality and poor treatment tolerability, both of which restrict durability of response and ultimately overall survival. One potential approach is to use host response biomarkers based on biochemical and haematological indices. Several such markers have been found to be associated with adverse outcomes, including inflammatory markers such as C-reactive protein (CRP),^11^ renal dysfunction,^12^ and the ratio of lymphocytes to total white blood cells.^13^ The use of laboratory markers to measure a patient's susceptibility to early mortality and treatment-related toxicity is objective and easy to implement with routinely measured parameters.

**Research in context**

**Evidence before this study**

Multiple myeloma is a disease that predominantly occurs in older patients in whom age-related physiological decompensation can lead to clinical frailty syndrome, which predisposes individuals to chronic disease, loss of independence, and mortality. Tolerability of anticancer therapy can impede treatment, affecting disease response and survival. Host-related factors have as much importance as tumour genomics in predicting outcomes in multiple myeloma. The International Myeloma Working Group (IMWG) published a Frailty Score in 2015 that predicts patient outcome, but this score has limitations in terms of the feasibility of implementing it in clinical practice and its objectivity. Before doing our study, we reviewed all publications citing the IMWG Frailty Score publication using a Web of Science Core Collection Cited Reference Search. This search was updated on Aug 1, 2018, and covered the period from the online publication of the IMWG Frailty Score on Jan 27, 2015. All abstracts were screened by DAC and K-LR and reviewed in detail by DAC.

**Added value of this study**

We have developed a risk profile which is quick to implement and objective for use in patients with multiple myeloma who are ineligible for stem-cell transplantation. The risk profile uses commonly collected clinical data and is simpler to use than the IMWG Frailty Score. We have shown that the risk profile is associated with clinical outcomes (overall survival, progression-free survival, and early mortality) as well as intended delivery of therapy (proportion of protocol dose delivered). The risk profile retained prognostic potential in patients treated with therapeutic agents with differing mechanisms of action—alkylating agent regimens, immunomodulatory triplets, and proteasome inhibitors—suggesting that its prognostic capabilities are treatment agnostic. The risk profile also remained prognostic across different cytogenetic risk groups, emphasising that in older patients, outcome is driven by both tumour and host biology.

**Implications of all the available evidence**

The ability of clinical scoring systems, such as that proposed here, to predict whether a patient is likely to stop treatment early because of treatment intolerability, could enable pre-emptive, upfront dose adjustments in patients with multiple myeloma, preventing toxicity and potentially enabling patients to stay on therapy for longer. This risk profile we developed could influence survival, but this hypothesis needs to be tested. None of the risk scoring systems previously developed in myeloma are dynamic, making them unable to accommodate changes in disease-related frailty that might be minimised by effective anti-myeloma therapy. There is therefore scope to improve clinical risk scores by the addition of a suitable frailty biomarker, which is currently still in developmental stages. The use of risk profiles to direct therapy is an exciting possibility that will be explored further in collaborative clinical trials (eg, the UK-MRA Myeloma XIV [FiTNEss] study).

The primary aim of this analysis was to develop and validate a scoring system based on readily available and routinely collected clinical and laboratory data that could predict overall survival in transplant-ineligible patients. Exploratory objectives were to analyse the groups derived from the scoring system for association with progression-free survival, early mortality, dose delivery, baseline patient-reported health-related quality of life, and the independence of outcome prediction within various subgroups of patients.

## Methods

### Study design and participants

All newly diagnosed patients recruited to the non-intensive treatment pathway of the National Cancer Research Institute Myeloma XI study (NCRI-XI, ISRCTN49407852) were considered for inclusion in the training dataset and all those included in the training dataset were also included in the internal validation dataset. Similarly, all newly diagnosed patients recruited to the Medical Research Council Myeloma IX study (MRC-IX, ISRCTN68454111) were considered for inclusion in the test dataset.

The NCRI-XI trial was a phase 3, open-label, parallel-group, multi-arm, adaptive design trial with three randomisation stages done at 110 National Health Service (NHS) hospitals in England, Scotland, and Wales. The MRC-IX trial was a phase 3, open-label, two-by-two factorial design trial with two randomisation stages done at 120 NHS hospitals in England, Northern Ireland, Scotland, and Wales. Inclusion and exclusion criteria were broadly similar in both studies. Eligible patients were aged 18 years and older, with symptomatic multiple myeloma based on bone marrow clonal plasma cells and related organ or tissue impairment. Exclusion criteria included previous or concurrent malignancies, including myelodysplastic syndromes, previous treatment for myeloma (except for local radiation therapy, bisphosphonates, and corticosteroids), grade 2 or higher peripheral neuropathy, acute renal failure (unresponsive to up to 72 h of rehydration, characterised by creatinine >500 μmol/L or urine output <400 mL per day or requiring dialysis), active or previous hepatitis C infection, or women who were pregnant or lactating. Patients were identified and recruited through local multidisciplinary team meetings.

All patients provided written informed consent. The studies were approved by the national ethics review board (National Research Ethics Service, UK), institutional review boards of the participating centres, and the competent regulatory authority (Medicines and Healthcare Products Regulatory Agency, UK), and done according to the Declaration of Helsinki and the principles of Good Clinical Practice as espoused in the Medicines for Human Use (Clinical Trials) Regulations.

In both trials, patients who were young and fit enough to tolerate autologous stem-cell transplantation (transplant-eligible) entered the intensive treatment pathway; these patients are not considered in this analysis. Older and less fit patients (transplant-ineligible) entered the non-intensive treatment pathway. Strict age limits were deliberately avoided so that fit, older patients could receive intensive therapy and autologous stem-cell transplantation. The decision of treatment pathway was made on an individual patient basis, taking into account performance status, clinician judgement, and patient preference.

Patients, clinicians, and the trial team were aware of the randomised allocation as the study was open-label. At the time of this analysis, the trial results of NCRI-XI were not published and so no analysis stratified by randomised allocation is reported here. The primary and secondary outcomes of the MRC-IX study have been published previously.^14^, ^15^, ^16^, ^17^, ^18^, ^19^

### Procedures

In the NCRI-XI study, transplant-ineligible patients were randomly assigned to receive attenuated-dose versions of the oral cyclophosphamide, thalidomide, and dexamethasone regimen (CTDa; cyclophosphamide 500 mg weekly, thalidomide initially 50 mg daily for 28 days and increasing every 28 days by 50 mg increments to 200 mg daily, and dexamethasone 20 mg daily on days 1–4 and 15–18) or the oral cyclophosphamide, lenalidomide, and dexamethasone induction regimen (CRDa; cyclophosphamide 500 mg on days 1 and 8, lenalidomide 25 mg daily for 21 days, and dexamethasone 20 mg daily on days 1–4 and 15–18). Treatment continued for at least six cycles in the absence of progressive disease, until maximum response or intolerance was observed. Patients randomly assigned to the immunomodulatory-based triplet regimens followed a response-adapted approach. Those with complete response or very good partial response proceeded directly to maintenance randomisation. All patients with partial response or minimal response were randomly assigned to receive up to eight cycles of cyclophosphamide, bortezomib, and dexamethasone (CVD; cyclophosphamide 500 mg on days 1, 8 and 15, bortezomib 1·3 mg/m^2^ subcutaneously or intravenously administered on days 1, 4, 8, and 11, and dexamethasone 20 mg daily on days 1–2, 4–5, 8–9, and 11–12) or no additional treatment before maintenance randomisation. Patients with less than minimal response all received up to eight cycles of CVD. For maintenance therapy, at maximum response for transplant-ineligible patients, patients were initially allocated to receive either lenalidomide 25 mg per day (orally on days 1–21 of each 28-day cycle) or observation. Following a protocol amendment on Sept 14, 2011, patients were allocated to receive lenalidomide 10 mg per day (orally on days 1–21 of each 28-day cycle), lenalidomide plus vorinostat, or observation within protocol version 5.0. A further protocol amendment to version 6.0 was implemented on June 28, 2013, where patients were allocated to receive lenalidomide 10 mg per day (orally on days 1–21 of each 28-day cycle) or observation, and the lenalidomide plus vorinostat group was discontinued because of withdrawal of the supply of vorinostat.

In the MRC-IX study, transplant-ineligible patients were randomly assigned to either oral melphalan and prednisone (7 mg/m^2^ melphalan and 40 mg prednisone, both on days 1–4) or to attenuated-dose versions of the oral cyclophosphamide, thalidomide, and dexamethasone regimen (CTDa; cyclophosphamide 500 mg weekly, thalidomide initially 50 mg daily for 28 days and increasing every 28 days by 50 mg increments to 200 mg daily, and dexamethasone 20 mg daily on days 1–4 and 15–18). Treatment continued for at least six cycles in the absence of progressive disease, until maximum response or intolerance was observed, up to a maximum of nine cycles. Patients were also assigned to bisphosphonate treatment (oral clodronic acid 1600 mg per day or intravenous zoledronic acid 4 mg every 21–28 days with induction chemotherapy, and every 28 days thereafter). After initial therapy, all eligible patients were randomly assigned to low-dose thalidomide maintenance therapy given until disease progression (50 mg daily for 28 days, increasing thereafter to 100 mg daily if well tolerated) or observation.

In both trials, response and progression were assessed according to International Myeloma Working Group (IMWG) Uniform Response criteria^18^, ^19^ and reviewed centrally by an expert panel, which was masked to treatment allocation.

For this study, as well as in the trials, patients were classified according to the presence of adverse risk cytogenetics, defined as gain(1q), t(4;14), t(14;16), t(14;20) and del(17p), as: genetic standard risk (no adverse lesions); genetic high risk (one adverse lesion); or genetic ultra-high risk (two or more adverse lesions)^20^ and also by use of the IMWG definition of adverse cytogenetic risk, defined as t(4;14), t(14;16) and del(17p), as: genetic standard risk (no adverse lesions) and genetic high risk (one or more adverse lesions).^21^, ^22^

### Outcomes

The primary endpoint used for the development and validation of the UK Myeloma Risk Alliance Risk Profile (MRP) was overall survival. Overall survival was defined as the time from randomisation to death from any cause. Otherwise, patients were censored at the time they were last known to be alive. Various exploratory endpoints were also analysed: progression-free survival, early mortality, percentage of protocol dose delivered (NCRI-XI only), and health-related quality of life (MRC-IX only). Progression-free survival was defined as the time from randomisation to the date of progression or death from any cause or censored at the time the patient was last known to be alive and progression free. Early mortality was defined as death within 60 days of randomisation (yes or no). Time-to-event endpoints for this analysis were censored at the date of database lock: Oct 28, 2016, in NCRI-XI and Jan 5, 2012, in MRC-IX. Data about the percentage of protocol dose delivered were available in NCRI-XI only and defined as the percentage delivered of the minimum protocol dose defined for induction treatment (six cycles of treatment) and the maximum protocol dose defined for consolidation treatment (eight cycles). Patient-reported health-related quality of life was available in MRC-IX only and was defined with the 20 subscales of the scored versions of the EORTC QLQ-C30^23^ and the EORTC QLQ-MY24 questionnaires.^24^, ^25^

### Statistical analysis

Patient characteristics and biochemical and haematological measurements, collected before treatment initiation, were considered potential prognostic variables. These were chosen as they were relatively easy to assess or were commonly available when patients presented. The potential prognostic variables included WHO performance status, lactate dehydrogenase (LDH), CRP, international staging system (ISS), age, and the ratio of lymphocytes to total white blood cells (L:W). WHO performance status (0–5) and ISS (I-III) were treated as ordinal variables and modelled with polynomial contrasts. LDH, CRP, age, and L:W were treated as continuous variables, where LDH was transformed with log transformations and CRP with (log+1) transformations. Complete case data in the training set (data from NCRI-XI) were defined as individuals having all potential prognostic variables recorded. Missing data were imputed via multiple imputation by chained equations to create ten imputed datasets.^26^

Continuous variables were standardised with the mean and SD within each imputed dataset. Preliminary investigations consisted of univariate Cox proportional hazards models, considering the association between overall survival and each individual potential prognostic variable, within each imputed dataset. Coefficients and standard errors were combined with Rubin's rules.^27^ A multivariable penalised Cox model was estimated to investigate the association between the potential prognostic variables and overall survival within each imputed dataset to give ten models.^28^ A least absolute shrinkage and selection operator penalty term was applied and the same penalty parameter was used within each imputed dataset. The penalty term was estimated as the average of the optimal values, estimated separately by cross-validation, within each imputed dataset. Standard errors for the coefficients from each model were obtained via bootstrap methods.^29^

The calibration of the ten models was assessed with predicted versus observed probability plots and the discrimination of the models was assessed with the prognostic separation D-statistic (D-statistic). For calibration, predicted versus observed probability plots were plotted at 60 days and 1 year after randomisation. The nature of censoring within survival data resulted in the predicted probability being calculated with the interval approach, similar to that described by Harrell and colleagues.^30^ To account for the full range of probabilities appropriately, ten intervals were chosen, similar to the calibration assessment done by Clark and Altman.^26^ The observed probabilities were calculated via the Kaplan-Meier method. The D-statistic is a measure of discrimination for time-to-event endpoints, of which higher values indicate better discrimination.^31^ The measure is essentially a log-hazard ratio from a Cox model—it can take any value on the real line and a value of zero is comparable to a hazard ratio of one in which there is no difference in two survival curves, or a value of approximately −0·693 is comparable to a hazard ratio of 0·5 and a value of approximately 0·693 is comparable to a hazard ratio of 2. The D-statistic has been successfully applied in the development and validation of various risk models^32^, ^33^, ^34^ and is acknowledged within the TRIPOD (transparent reporting of a multivariable prediction model for individual prognosis or diagnosis) reporting guidelines as an appropriate measure of discrimination.^35^

The MRP was obtained by combining the estimates from the ten models using Rubin's rules. It was assessed by summarising the calibration results across the ten imputed datasets with the median and IQR and combining the discrimination results by use of Rubin's rules.^36^ This combination of the discrimination results gave the combined D-statistic.

The MRP was internally validated with the D-statistic within a bootstrap validation process,^30^ while accounting for imputation.^37^ A validation rule was prespecified as follows: if the corrected D-statistic lay within the 95% CI for the combined D-statistic then the MRP was concluded to be internally validated. The MRP was retrospectively externally validated with relevant complete case data from the test dataset (data from MRC-IX), where an individual was defined to have relevant complete case data if they had all the variables included in the MRP recorded. Variables in MRC-IX were standardised identically to NCRI-XI. External validation used the coefficients from the MRP to obtain the prognostic index (linear predictor), from which the D-statistic was estimated. An external validation rule was also prespecified: if the 95% CI for the D-statistic in MRC-IX overlapped with the 95% CI for the combined D-statistic in NCRI-XI then the MRP would be concluded to be externally validated.

The prognostic index of the MRP was obtained by combining the prognostic indices of the models estimated within the ten imputed datasets with Rubin's rules. The tertiles of this combined prognostic index were used to trichotomise individuals into the MRP groups: low risk, medium risk, and high risk. The same bounds were applied in MRC-IX to obtain a similar division of patients. The distributions of the prognostic variables considered were compared across the MRP groups to assess their clinical appropriateness. The MRP groups were then used to stratify overall survival with Kaplan-Meier estimates of the survivor function. The discrimination of this model was assessed with the grouped version of the D-statistic. Exploratory and subgroups analyses are described in the appendix (p 2).

Statistical analyses were done in SAS, version 9.4, and R.

### Role of the funding source

The funders had no role in study design, data analysis, data interpretation, or writing of the manuscript. All authors had full access to all the data in the study and had final responsibility for the decision to submit for publication. The authors are solely responsible for study design, data collection, data analysis, data interpretation, writing of the report, and decisions about submission for publication.

## Results

**Figure 1:**
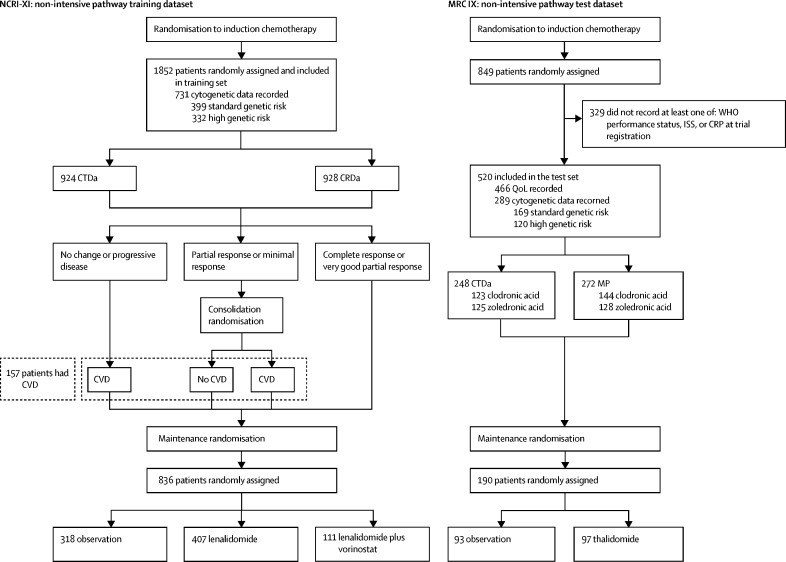
Non-intensive pathways of the NCRI Myeloma XI and MRC Myeloma IX studies Standard genetic risk defined as the absence of any of the risk lesions t(4;14), t(14;26), t(14;20), del(17p) and gain(1q); high genetic risk defined as the presence of at least one of these lesions. CRDa=attenuated cyclophosphamide, lenalidomide, and dexamethasone. CRP=C-reactive protein. CTDa=attenuated cyclophosphamide, thalidomide, and dexamethasone. CVD=cyclophosphamide, bortezomib, and dexamethasone. ISS=International Staging System. MP=melphalan and prednisolone. MRC-IX=MRC Myeloma IX. NCRI-XI=NCRI Myeloma XI. QoL=quality of life.

The NCRI-XI study recruited 1852 previously untreated transplant-ineligible patients between May 25, 2010, and April 20, 2016, with a median age of 74 years (IQR 71–78). 322 (17%) of 1852 trial participants were aged 80 years or older (table 1). All patients in the NCRI-XI study formed the training dataset, of whom 1268 (68%) had complete case data. The non-intensive pathway of the MRC-IX trial recruited 849 patients between May 14, 2003, and Nov 20, 2007, of whom 520 (61%) had complete case data and were included in the test dataset. Figure 1 shows the constituents of the test and training datasets from these trials, and the number of patients included in the various subgroups for exploratory analyses, with further details provided in the appendix (p 2). Patients in MRC-IX were slightly younger (933 [50%] of 1852 aged <75 years in NCRI-XI *vs* 318 [61%] of 520 in MRC-IX). Other baseline variables such as WHO performance status and ISS were similar (table 1). For the primary endpoint of overall survival, 700 (38%) of 1852 patients in NCRI-XI and 411 (79%) of 520 in MRC-IX had died at the time of analysis. For progression-free survival, 1273 (69%) of 1852 patients had an event in NCRI-XI, as did 500 (96%) of 520 in MRC-IX. For early mortality, 83 (4%) of 1852 patients died within 60 days of randomisation in NCRI-XI, as did 36 (7%) of 520 in MRC-IX.

**Table 1:** Baseline characteristics and treatment details for the training and test datasets

		**NCRI-XI: training set**	**MRC-IX: test set**		
		Included (n=1852)	Included (n=520)	Excluded (n=329)	Total (n=849)
Median age		74·0 (71·0–78·0)	73·0 (70·0–77·0)	74·0 (70·0–77·0)	73·0 (70·0–77·0)
Age group (years)					
	<70	313 (17%)	129 (25%)	81 (25%)	210 (25%)
	70–74	620 (34%)	189 (36%)	108 (33%)	297 (35%)
	75–79	597 (32%)	131 (25%)	103 (31%)	234 (28%)
	≥80	322 (17%)	71 (14%)	37 (11%)	108 (13%)
Sex					
	Men	1047 (57%)	285 (55%)	188 (57%)	473 (56%)
	Women	805 (43%)	235 (45%)	141 (43%)	376 (44%)
WHO performance status					
	0	478 (26%)	93 (18%)	63 (19%)	156 (18%)
	1	810 (44%)	238 (46%)	171 (52%)	409 (48%)
	2	342 (18%)	111 (21%)	62 (19%)	173 (20%)
	3	110 (6%)	68 (13%)	28 (9%)	96 (11%)
	4	11 (1%)	10 (2%)	1 (0%)	11 (1%)
	Data missing	101 (6%)	0 (0%)	4 (1%)	4 (1%)
ISS stage					
	I	318 (17%)	68 (13%)	42 (13%)	110 (13%)
	II	731 (39%)	215 (41%)	97 (29%)	312 (37%)
	III	659 (36%)	237 (46%)	96 (29%)	333 (39%)
	Data missing	144 (8%)	0 (0%)	94 (29%)	94 (11%)
CRP (mg/L)		5·0 (3·0–16·0)	8·0 (4·0–20·0)	7·0 (5·0–19·0)	8·0 (4·0–20·0)
	Data missing	262	0	276	276
LDH (IU/L)		278·0 (190·0–396·5)	328·0 (244·0–423·0)	324·0 (230·0–433·0)	328·0 (242·5–424·0)
	Data missing	416	159	218	377
L:W		0·3 (0·2–0·4)	0·3 (0·2–0·3)	0·3 (0·2–0·4)	0·3 (0·2–0·4)
	Data missing	7	2	2	4
Cytogenetic risk groups					
	Standard	399 (22%)	169 (33%)	84 (26%)	253 (30%)
	High risk	265 (14%)	77 (15%)	52 (16%)	129 (15%)
	Ultra-high risk	67 (4%)	43 (8%)	14 (4%)	57 (7%)
	NA	1121 (61%)	231 (44%)	179 (54%)	410 (48%)
Induction regimen					
	CTDa	924 (50%)	248 (48%)	178 (54%)	426 (50%)
	CRDa	928 (50%)	NA	NA	NA
	MP	NA	272 (52%)	151 (46%)	423 (50%)
Allocated bisphosphonate					
	Sodium clodronate	NA	267 (51%)	156 (47%)	423 (50%)
	Zoledronic acid	NA	253 (49%)	173 (53%)	426 (50%)
CVD received					
	Yes	157 (8%)	NA	NA	NA
	No	1695 (92%)	NA	NA	NA
Maintenance regimen					
	Observation	318 (17%)	93 (18%)	70 (21%)	163 (19%)
	Thalidomide	NA	97 (19%)	66 (20%)	163 (19%)
	Lenalidomide	407 (22%)	NA	NA	NA
	Lenalidomide and vorinostat	111 (6%)	NA	NA	NA
	No maintenance regimen	1016 (55%)	330 (63%)	193 (59%)	523 (62%)

Data are n (%) or median (IQR). NCRI-XI=NCRI Myeloma XI. MRC-IX=MRC Myeloma IX. ISS=International Staging System. CRP=C-reactive protein. LDH=lactate dehydrogenase. L:W=lymphocyte to total white blood cell ratio. NA=not applicable. CTDa=attenuated cyclophosphamide, thalidomide, and dexamethasone. CRDa=attenuated cyclophosphamide, lenalidomide, and dexamethasone. MP=melphalan and prednisolone. CVD=cyclophosphamide, bortezomib, and dexamethasone. Standard risk was defined as the absence of any of the risk lesions: t(4;14), t(14;16), t(14;20), del(17p) and gain(1q). High risk was defined as one lesion present. Ultra-high risk was defined as more than one lesion present (UK definition). High risk and ultra-high risk categories were combined and defined as high risk for the analysis.

The univariate Cox model showed that age, L:W, CRP, and the linear element of WHO performance status and the linear element of ISS were significantly associated with overall survival (table 2). The MRP included the linear and quadratic terms of WHO performance status, the linear term of ISS, age, and CRP, demonstrating that WHO performance status, ISS, age, and CRP are associated with overall survival in a multivariable analysis (table 2). The calibration and discrimination of the MRP were good. Comparing calibration at 60 days after randomisation (appendix p 3) to calibration at 1 year after randomisation (appendix p 3) showed that the model is better calibrated closer in time to baseline, as the intervals for each risk group intersect with the desired line representing perfect calibration for 60 days after randomisation compared with 1 year after randomisation. The combined D-statistic for the MRP was 0·840 (95% CI 0·718–0·963), demonstrating good discrimination of outcome. The corrected D-statistic from the internal validation process was 0·801 and the D-statistic for the MRC-IX model was 0·654 (95% CI 0·497–0·811). The inclusion of the corrected D-statistic and the overlap between confidence intervals demonstrates that the MRP was successfully internally and retrospectively externally validated according to prespecified criteria.

**Table 2:** Univariate Cox regression and multivariable penalised Cox regression model results for overall survival within the training dataset (NCRI-XI)

		**Univariate model results**			**Multivariable model results**		
		Coefficient (SE)	Hazard ratio (SE)	95% CI for HR	Coefficient* (SE)	Hazard ratio (SE)	95% CI for HR
WHO performance status							
	Linear	0·794 (0·269)	2·211 (1·309)	(1·305–3·747)	0·629 (0·171)	1·875 (1·187)	(1·340–2·624)
	Quadratic	−0·409 (0·231)	0·664 (1·260)	(0·422–1·044)	−0·001 (0·076)	0·999 (1·079)	(0·861–1·160)
	Cubic	−0·098 (0·160)	0·907 (1·173)	(0·663–1·240)	0 (0)	1 (1)	1 (1–1)
	Quartic	−0·027 (0·100)	0·973 (1·105)	(0·800–1·184)	0 (0·001)	1 (1·001)	(0·999–1·001)
Standardised age		0·260 (0·040)	1·296 (1·041)	(1·198–1·402)	0·089 (0·040)	1·093 (1·041)	(1·010–1·183)
ISS							
	Linear	0·617 (0·088)	1·853 (1·092)	(1·560–2·201)	0·299 (0·075)	1·349 (1·078)	(1·165–1·562)
	Quadratic	0·080 (0·073)	1·084 (1·076)	(0·940–1·250)	0 (0·015)	1 (1·015)	(0·971–1·029)
Standardised L:W		−0·180 (0·048)	0·835 (1·049)	(0·760–0·917)	0 (0·007)	1 (1·007)	(0·987–1·013)
Standardised transformed CRP		0·241 (0·038)	1·273 (1·038)	(1·182–1·370)	0·035 (0·034)	1·036 (1·034)	(0·970–1·106)
Standardised transformed LDH		0·054 (0·044)	1·055 (1·044)	(0·969–1·149)	0 (0)	1 (1)	(0·999–1·001)

CRP=C-reactive protein. HR=hazard ratio. ISS=International Staging System. L:W=lymphocyte to total white blood cell ratio. LDH=lactate dehydrogenase. NCRI-XI=NCRI Myeloma XI.

* The coefficients marked as zero have been penalised during the model building process and are therefore not included in the UK Myeloma Research Alliance Risk Profile.

**Figure 2:**
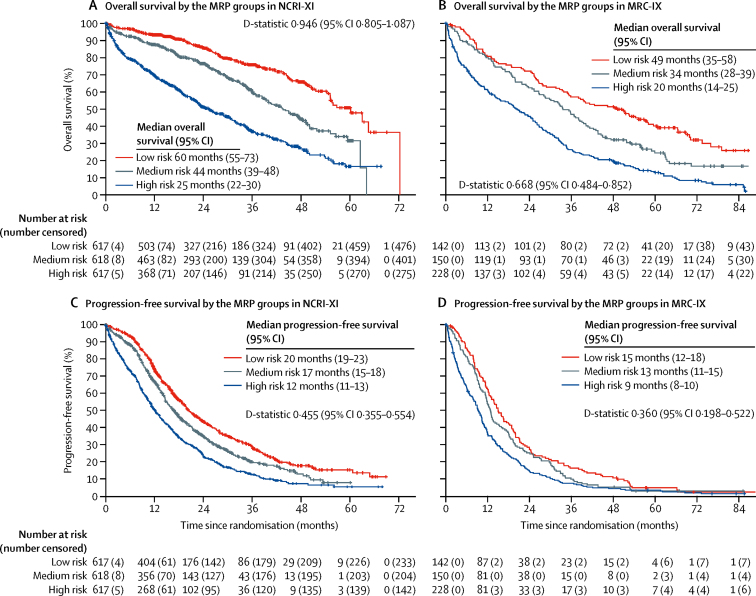
Kaplan-Meier curves for overall survival and progression-free survival across the MRP groups (A) Overall survival in the training population, NCRI Myeloma XI (NCRI-XI; n=1852). (B) Overall survival in the test population, MRC Myeloma IX (MRC-IX; n=520). (C) Progression-free survival in NCRI-XI (n=1852). (D) Progression-free survival in MRC-IX (n=520). MRP=UK Myeloma Research Alliance Risk Profile.

The MRP scoring algorithm (appendix p 4) categorises individuals into groups: low, medium, and high risk, where the cutoff points represent the tertiles of the combined prognostic index (33%: −0·256; 66%: −0·0283). Each of the variables considered as prognostic factors increased in severity across the three groups, in both MRC-IX and NCRI-XI (appendix p 10). The MRP groups were associated with overall survival in both NCRI-XI (figure 2A) and MRC-IX (figure 2B).

The MRP groups were also associated with progression-free survival in NCRI-XI (figure 2C) and MRC-IX (figure 2D).

The discrimination performance of the MRP groups for progression-free survival was acceptable because of the overlapping confidence intervals for the D-statistic (NCRI-XI D-statistic 0·455 [95% CI 0·355–0·554], MRC-IX D-statistic 0·360 [0·198–0·522]).

The MRP groups predicted early mortality in both studies. The area under the receiver operating characteristic curve was 0·720 (95% CI 0·657–0·783) for the model within NCRI-XI and 0·660 (0·607–0·713) for the model within MRC-IX, demonstrating that the MRP groups had good discriminative ability. The odds ratio for early mortality compared with low-risk patients was 2·14 (95% CI 1·04–4·42) for medium-risk patients and 4·76 (2·44–9·27) for high-risk patients in NCRI-XI and 1·92 (95% CI 0·35–10·54) for medium-risk patients and 10·59 (2·48–45·17) for high-risk patients in MRC-IX (appendix p 10).

**Figure 3:**
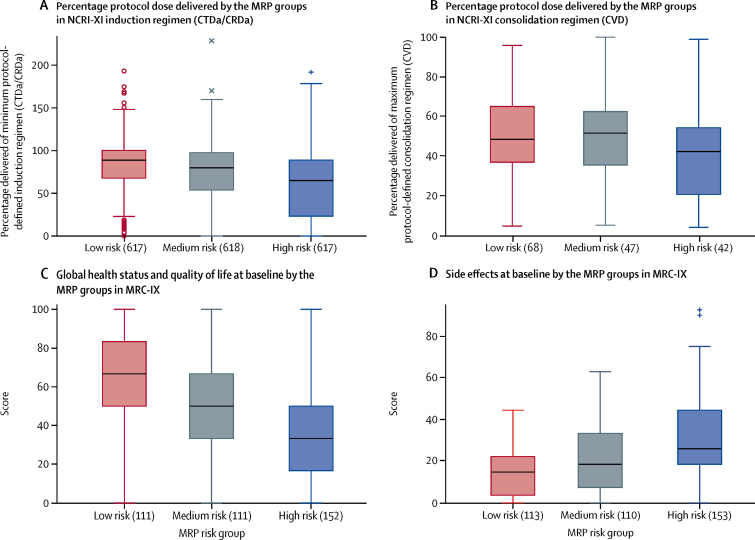
Box-plots showing percentage of protocol dose delivered in NCRI-XI and EORTC QLQ-C30 subscale scores in MRC-IX across the MRP groups (A) Percentage of protocol dose delivered in NCRI Myeloma XI (NCRI-XI; n=1852) for induction regimen. (B) Percentage of protocol dose delivered in NCRI-XI (n=157) for consolidation regimen. (C) EORTC QLQ-C30 subscale scores in MRC Myeloma IX (MRC-IX; n=466) for global health status/quality of life. Higher values indicate improved response. (D) EORTC QLQ-C30 subscale scores in MRC-IX for side-effects of treatment measured at baseline. Higher values indicate worse response. All data are median (IQR). Symbols (round circles, +, and ×) indicate outliers. CRDa=attenuated cyclophosphamide, lenalidomide, and dexamethasone. CTDa= attenuated cyclophosphamide, thalidomide, and dexamethasone. CVD=cyclophosphamide, bortezomib, and dexamethasone. MRP=UK Myeloma Research Alliance Risk Profile.

The percentage of protocol dose delivered in NCRI-XI was examined across the MRP groups to investigate the association between the risk profile and treatment tolerability. The median percentage of protocol dose delivered of induction therapy reduced as the risk to individuals increased (figure 3A). During induction, low-risk patients received 88·57% (95% CI 67·53–100) of the minimum protocol dose, while medium-risk patients received 79·63% (53·98–97·22), and high-risk patients 64·79% (23·28–88·69). This reduction is less marked in the CVD consolidation phase (figure 3B) where the percentage of protocol dose delivered was similar across all MRP groups, with no notable per-drug variability. This might have been due to the fact that only patients able to tolerate completion of initial induction therapy and judged to be able to tolerate the planned infusion therapy reached consolidation. The percentage of protocol dose delivered appears to be lower for consolidation than for induction overall, but this is due to the protocol stipulation of a minimum number of cycles for induction compared to a maximum number of cycles for consolidation.

Health-related patient reported quality-of-life subscales, measured at baseline, were compared across the MRP groups in MRC-IX. Of the 520 individuals in the test dataset, 466 (90%) had quality of life recorded at baseline. There was a clear association between worsening subscale scores and increase in risk, supporting stratification of patients with the MRP groups (figures 3C, D; appendix pp 5,6). For example, the median global health status/quality-of-life subscale from the EORTC QLQ-C30 instrument decreased step-wise between low risk (66·7, IQR 50·0–83·3), medium risk (50·0, 33·3–66·7), and high risk (33·3, 16·7–50·0) groups. A similar association between worsening response and increase in risk was observed with the side-effects of treatment subscale from the EORTC QLQ-MY24 module.

**Figure 4:**
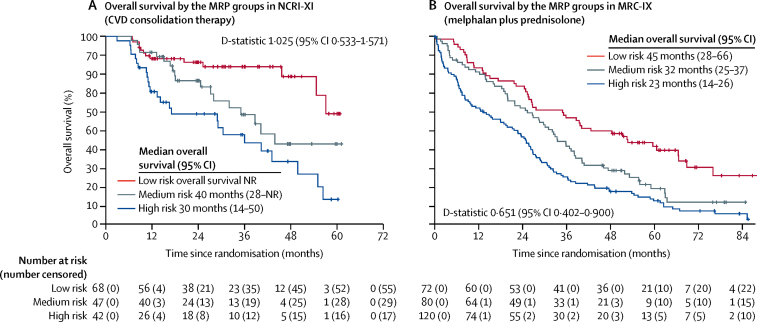
Kaplan-Meier curves for overall survival by MRP groups in patients receiving combinations without immunomodulatory drugs (A) CVD consolidation therapy in NCRI Myeloma XI (NCRI-XI; n=157). (B) Melphalan plus prednisolone in MRC Myeloma IX (MRC-IX; n=272). CVD=cyclophosphamide, bortezomib, and dexamethasone. MRP=UK Myeloma Research Alliance Risk Profile. NR=not reached.

Although derived in patients receiving the immuno-modulatory agent containing regimens in NCRI-XI, the MRP groups remained prognostic for the 272 patients exposed to alkylator chemotherapy alone (melphalan plus prednisolone in MRC-IX) and in the 157 patients exposed to sequential immunomodulatory and proteasome inhibitor therapy (CVD consolidation in NCRI-XI; figure 4). The discriminatory power of the MRP groups was therefore not specific to the class of therapy used.

**Figure 5:**
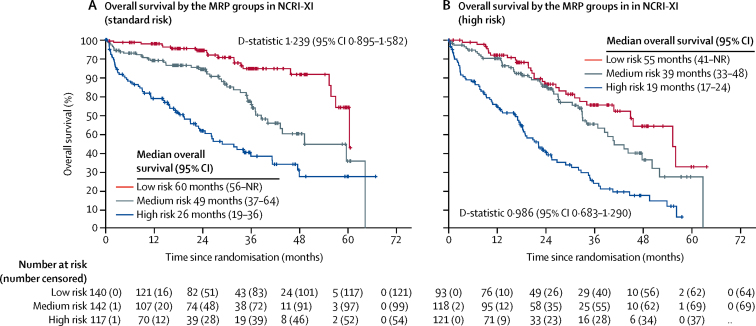
Kaplan-Meier curves for overall survival for patients with genetic standard and high risk profiles across the MRP groups (A) Patients in NCRI Myeloma XI (NCRI-XI) with standard cytogenetic profiles (n=399). B) Patients in NCRI-XI with high-risk cytogenetic profiles (n=332). Standard risk defined as the absence of any of the risk lesions; t(4;14), t(14;16), t(14;20), del(17p) and gain(1q); high risk defined as the presence of at least one risk lesion (UK definition). MRP=UK Myeloma Research Alliance Risk Profile. NR=not reached.

The MRP also remained prognostic within the cytogenetic risk groups (genetic standard and genetic high-risk) in both datasets according to the UK definition (NCRI-XI: n=731 [399 genetic standard risk, 332 genetic high-risk], figure 5; MRC-IX: n=289 [169 genetic standard risk, 120 genetic high-risk], appendix p 7) and according to the IMWG definition (MRC-IX: n=289 [231 genetic standard risk, 58 genetic high-risk]; NCRI-XI: n=731 [598 genetic standard risk, 133 genetic high-risk] appendix pp 8,9). In all cases there was a similar discriminative performance to that observed overall, demonstrating the ability of the MRP group to determine outcome based on host biology, independently of tumour cell aggressiveness.

## Discussion

Using data from two of the largest clinical trials done in newly diagnosed transplant-ineligible patients with multiple myeloma, we have derived a novel risk profile that appears to be prognostic of overall survival. The score uses prognostic factors regularly measured at baseline: laboratory blood tests (ISS and CRP), age, and a simple WHO performance status assessment, and therefore does not require any additional investigations or assessments in addition to standard of care. The risk profile has been successfully validated, internally and externally, according to predefined criteria. The risk profile is associated with progression-free survival, early mortality, and the percentage of protocol dose delivered. It is also associated with baseline quality-of-life subscales from commonly used patient-reported outcome measures. The risk profile also retains prognostic potential in patients treated with different mechanisms of action: alkylating agent regimens (eg, melphalan plus prednisolone), immunomodulatory triplets (eg, CTDa or CRDa) and in patients treated with a sequential combination of immunomodulatory agents and proteasome inhibitors (eg, CTDa or CRDa plus CVD). This indicates that this risk profile can be applied to patients treated with most therapeutic agents currently available in routine clinical practice for transplant-ineligible patients with myeloma. Importantly, the risk profile is independently prognostic in genetic high-risk patients according to both the UK definition and the IMWG definition (used in defining the revised ISS), suggesting that in older patients the outcome is driven by both tumour and host biology.

The reference model with which the risk profile should be compared is the validated IMWG Frailty Score.^8^, ^9^ This score requires completion of three questionnaires: the ADL,^5^ IADL,^6^ and the CCI.^7^ Such instruments potentially incorporate a degree of subjectivity, and can be open to bias. Additionally, the IMWG score remains influenced by age cutoffs, with all patients older than 80 years classified as frail. However, a limitation of this report is that in the NCRI-XI and MRC-IX trials these questionnaires were not administered and therefore a direct comparison is not possible. However, the MRP will be prospectively validated and compared with the IMWG Frailty Score in the UK-MRA Myeloma XIV study (FiTNEss), which will begin in June, 2019, incorporating treatment strategies with the modern proteasome inhibitor ixazomib and the immunomodulatory agent lenalidomide in newly diagnosed transplant-ineligible patients with myeloma. Until such prospective validation is completed, the risk profile should only be used for research purposes.

The risk profile does not include adverse risk cytogenetics as a prognostic variable, although these have been shown by various academic groups to be important predictors of outcome in patients with myeloma. The definition of adverse risk cytogenetics differs between countries. For example, the UK defines t(4;14), t(14;16), t(14;20), del(17p) and gain(1q) as high-risk lesions,^20^ whereas the IMWG include only a subset of these,^21^, ^22^ and others have also proposed inclusion of a high-risk gene expression profile.^38^ We have shown that the risk profile remains prognostic in genetic high-risk patients according to either the UK or IMWG definition and that the effect of the risk profile is both independent and additive (figure 5; appendix pp 5,6). We believe that, although there is currently no consensus concerning genetic high-risk disease, including such terms in a risk profile for older patients would not be useful as it might limit future applicability in the UK and worldwide.

The risk profile was developed in patients from clinical trials, which could be considered a limitation for generalisability since clinical trials have stringent eligibility criteria—patients enrolled in these trials are different from those in the general population. Consequently, all risk profiles developed from clinical trial data, including that described here, might not necessarily provide outcome predictions that are representative of the general population. However, we do not believe this to be the case in our analysis, as the NCRI-XI and MRC-IX trials had relatively few exclusion criteria, recruited approximately 1000 patients per year at peak recruitment from a variety of centres, representing more than 30% of newly diagnosed patients with myeloma in the UK, and recruited a substantial proportion of patients aged older than 80 years, making them representative of the general population. Additionally, to further mitigate this limitation the risk profile will be externally validated in the MMRF CoMMpass Study, which includes real-world patients from Canada, Italy, Spain, and the USA.

Some of the treatment delivered in the trials used to develop and validate the risk profile could be considered to be old standard of care (eg, the melphalan plus prednisolone regimen and CTDa regimen) and perhaps suboptimal treatment options that might have negatively affected the outcome of patients. These treatments, and even the first-generation immunomodulatory agent thalidomide, are prescribed in various European countries and throughout the world, as shown in a recent summary of standard practice.^39^ However, a large proportion of patients in NCRI-XI, in whom the risk profile was derived, were receiving lenalidomide-based induction or maintenance, or both, and some patients also received consolidation treatment with the proteasome inhibitor bortezomib. Lenalidomide-based induction is a current standard-of-care regimen worldwide for transplant-ineligible patients following the results of the FIRST study.^40^ The score remained prognostic in each of these treatment subgroups (data not shown).

The overall calibration of the MRP declines temporally further from trial entry. This might be due to the use of variables recorded at baseline, which could change over time. For example, if they are disease driven, CRP and WHO performance status could improve as the patient responds to treatment. Alternatively, these prognostic factors could worsen over time if they are driven by age or general health, or both.

There are numerous prognostic factors in myeloma that can be considered to be related to tumour biology, tumour burden, or host factors. These have been combined into staging systems and prognostic models that differ in the variables they select as important, which could be related to the different biological subgroups they are being informal surrogates for. The mSMART risk stratification guidelines include tumour biology factors and ignore tumour burden or host-related factors, which are important in older patients.^41^ In previous studies, frailty and outcome have been associated with activities of daily living, comorbidities, and age; the association with comorbidities has been demonstrated widely. For example, a population-based study demonstrated that comorbidity is more common in patients with myeloma than in the general population and is greater in patients older than 65 years.^42^ Different generic and myeloma-specific comorbidity measures have been central to the development of the Revised Myeloma Comorbidity Index (R-MCI),^10^ which combines frailty assessment with renal and lung function assessments, age, and Karnofsky performance status. The R-MCI is suggested as an alternative to the IMWG frailty score^10^ that should be evaluated by IMWG and European Myeloma Network experts prospectively. We consider the MRP to be a further valid, and potentially less burdensome, alternative that will be prospectively evaluated.

Other proposed prognostic models have added laboratory measurements such as the concentration of N-terminal fragment of the type-B natriuretic peptide to simple factors such as age and WHO performance status to seek simple alternatives to the IMWG.^43^ The inclusion of such a biomarker can reflect biological subgroups that might have cardiac and renal disease. ^43^ Although arguably less specific, CRP might give similar indications concerning biological subgroups, which could be complementary to the clinical and demographic variables in the MRP. We propose that laboratory measurements such as these could provide prognostic potential as well as geriatric assessment and deserve further study. The use of a biomarker-driven score in this setting could provide an easier system for clinicians to use with reduced possibility of inter-observer variability.

The standard approach to therapy in transplant-ineligible patients with myeloma, and within each of the studies used to develop the risk profile, is to administer chemotherapy at full dose with a possible dose reduction for steroids, followed by reactive dose adjustments in the event of toxicities. The association of MRP groups with the percentage of protocol dose delivered suggests that the MRP might be able to predict early treatment cessation, which could enable pre-emptive, up-front dose adjustment of patients, preventing toxicity and potentially enabling patients to stay on therapy for longer. This possibility will be explored further alongside prospective validation of the risk profile in the UK-MRA Myeloma XIV (FiTNEss) study.

## Data sharing

Any requests for de-identified trial data and supporting material (data dictionary, protocol, and statistical analysis plan) will be reviewed by the respective trial management group in the first instance. Only requests that have a methodologically sound proposal and whose proposed use of the data has been approved by the trial's independent trial steering committee will be considered. Proposals should be directed to the corresponding author in the first instance; to gain access, data requestors will need to sign a data access agreement.
