# Supplementary material for: A clinical prediction model for outcome and therapy delivery in transplant-ineligible patients with myeloma (UK Myeloma Research Alliance Risk Profile): a development and validation study
Source: Lancet Haematol. 2019 Feb 6;6(3):e154–66. doi: 10.1016/S2352-3026(18)30220-5 (PMC6391517; doi:10.1016/S2352-3026(18)30220-5)
Supplement: Supplementary appendix [file mmc1.pdf]

THE LANCET
Haematology

Supplementary appendix

This appendix formed part of the original submission and has been peer reviewed. We post it as supplied by the authors.

Supplement to: Cook G, Royle K-L, Pawlyn C, et al. A clinical prediction model for outcome and therapy delivery in transplant-ineligible patients with myeloma (UK Myeloma Research Alliance Risk Profile): a development and validation study. *Lancet Haematol* 2019; published online Feb 6. http://dx.doi.org/10.1016/S2352-3026(18)30220-5.

Supplementary Appendix: A clinical prediction model for outcome and therapy delivery in transplant-ineligible patients with myeloma (UK Myeloma Research Alliance Risk Profile): a development and validation study

Contents

Supplementary Methods 2

Cytogenetic Profiling 2

Imputation of missing data 2

Use of the D-statistic 2

Exploratory Analyses 2

Supplementary Figures 3

Supplementary Figure 1: Combined calibration results at A) sixty days and B) one year. 3

Supplementary Figure 2: MRP Scoring Algorithm 4

Supplementary Figure 3: The distributions of the MRP groups for the remaining Quality of Life subscales of the EORTC QLQ-C30 and QLQ-MY24 in MRC-IX at baseline. 5

Supplementary Figure 4: Kaplan-Meier Curves for overall survival (OS) by the MRP groups in MRC-IX with A) Standard (n=169), B) High-Risk (n=120) cytogenetic profiles. Standard risk was defined as the absence of any of the risk lesions; t(4;14), t(14; 16), t(14;20), del(17p) and gain(1q), high-risk at least one risk lesion (UK definition). 7

Supplementary Figure 5: Kaplan-Meier curves for overall survival by the MRP groups for the patients in MRC-IX with A) Standard (n=231), B) High-Risk (n=58) cytogenetic profiles and patients in NCRI-XI with C) Standard (n=598) and D) High-Risk (n=133). Standard risk was defined as the absence of any of the risk lesions; t(4;14), t(14;16), and del(17p) high-risk at least one risk lesion (IMWG definition). 8

Supplementary Tables 10

Supplementary Table 1: Distribution of each potential prognostic factor across the MRP groups in the training (NCRI-XI) and test (MRC-IX) datasets 10

Supplementary Table 2: Logistic regression models for early mortality 10

References 11

Supplementary Methods

Cytogenetic Profiling

Diagnostic bone marrow samples were initially purified for plasma cells using CD138-based cell selection (Miltenyi Biotec, Bisley, UK). [1] In MRC Myeloma IX (MRC-IX) cytogenetic profiling was performed using fluorescence in situ hybridisation (FISH). In NCRI Myeloma XI (NCRI-XI) cytogenetic profiling was performed using Multiplex Ligation-dependent Probe Amplification (MLPA) and quantitative real-time PCR (qRT-PCR) on CD138+ve cells. [2,3]

Imputation of missing data

The ordinal and continuous variables were imputed using ordinal logistic and multivariable linear regression, respectively. Each imputation model included the other five prognostic factors, sex, overall survival time in months and an indicator as to whether the survival time related to an event (death) or a censored observation.

Use of the D-statistic

The D-statistic is obtained by regressing the outcome of interest on the rankits associated with the prognostic index (linear predictor) of the risk model. The rankits are derived by first ordering the prognostic index from smallest to largest and then calculating $\frac{1}{\sqrt{8/\pi}}\Phi^{-1}\left(\frac{i-3/8}{n+1/4}\right)$ for i=1 to n, where n represents the sample size used to create the risk model and Φ^{-1} the quantile function of the standard normal distribution. The quantile function gives a value at which the probability of the random variable taking that value is less than or equal to the probability stated within the function e.g. $\Phi^{-1}(0.5) = 0$ as the standard normal is centred at zero. The ith rankit then replaces the ith largest prognostic index and the rankits used within the regression model.

Exploratory Analyses

Applicability to other endpoints

Exploratory analysis examined the MRP groups' relationship to progression-free survival, early mortality, the percentage of protocol dose delivered and quality of life.. The survival functions for progression-free survival were estimated using the Kaplan-Meier method and the discrimination assessed using the grouped version of the D-statistic and informally compared to the grouped versions calculated for overall survival. Early mortality was analyzed using a logistic regression model and the area under the receiver operating characteristic curve (AUC) estimated as a measure of discrimination. The percentage of protocol dose delivered and quality of life at baseline were considered descriptively by comparing the relevant distributions across the MRP groups.

Subgroup analysis

The predictive ability of the MRP groups within subgroups of patients in terms of overall survival was assessed descriptively. The induction treatments (MRC-IX: CTDa and melphalan and prednisone, NCRI-XI: CTDa and CRDa), the consolidation therapy in NCRI-XI (CVD) and the cytogenetic risk groups in both studies (standard risk and high-risk) using the definition of high-risk cytogenetics according to the UK and the IMWG.

Supplementary Figures

Supplementary Figure 1: Combined calibration results at A) sixty days and B) one year.

A)

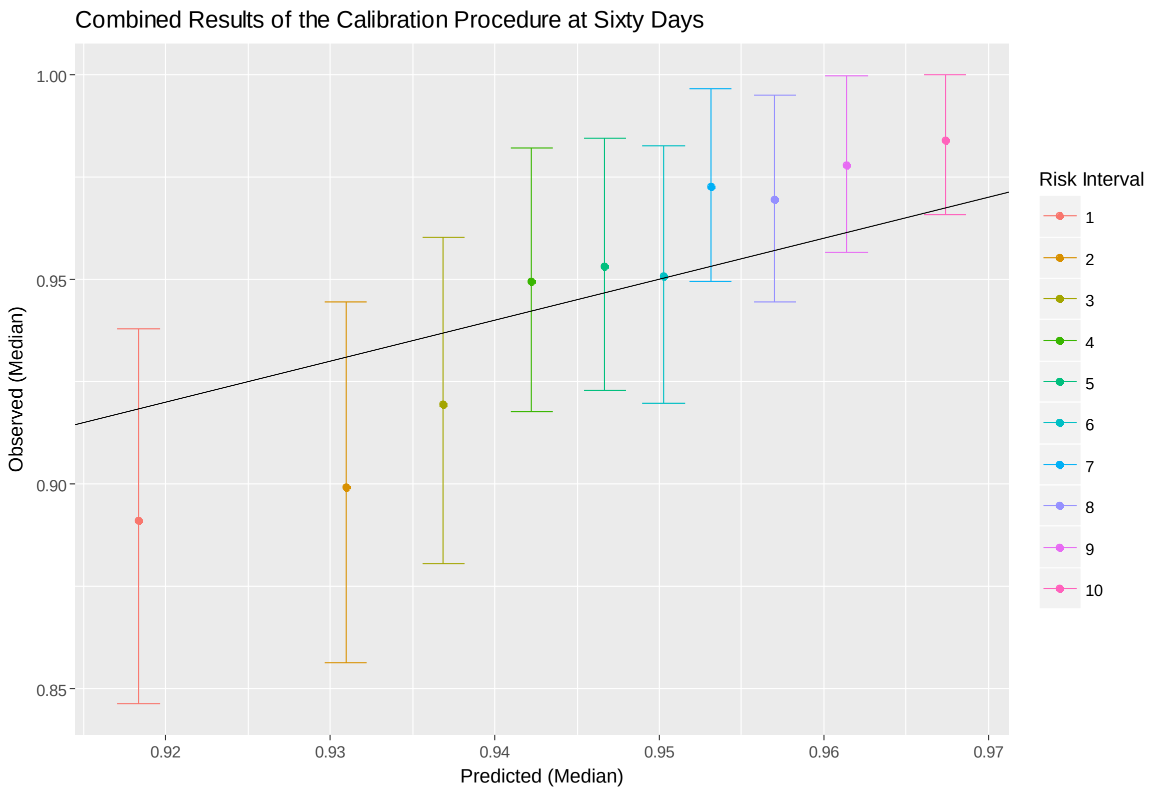

B)

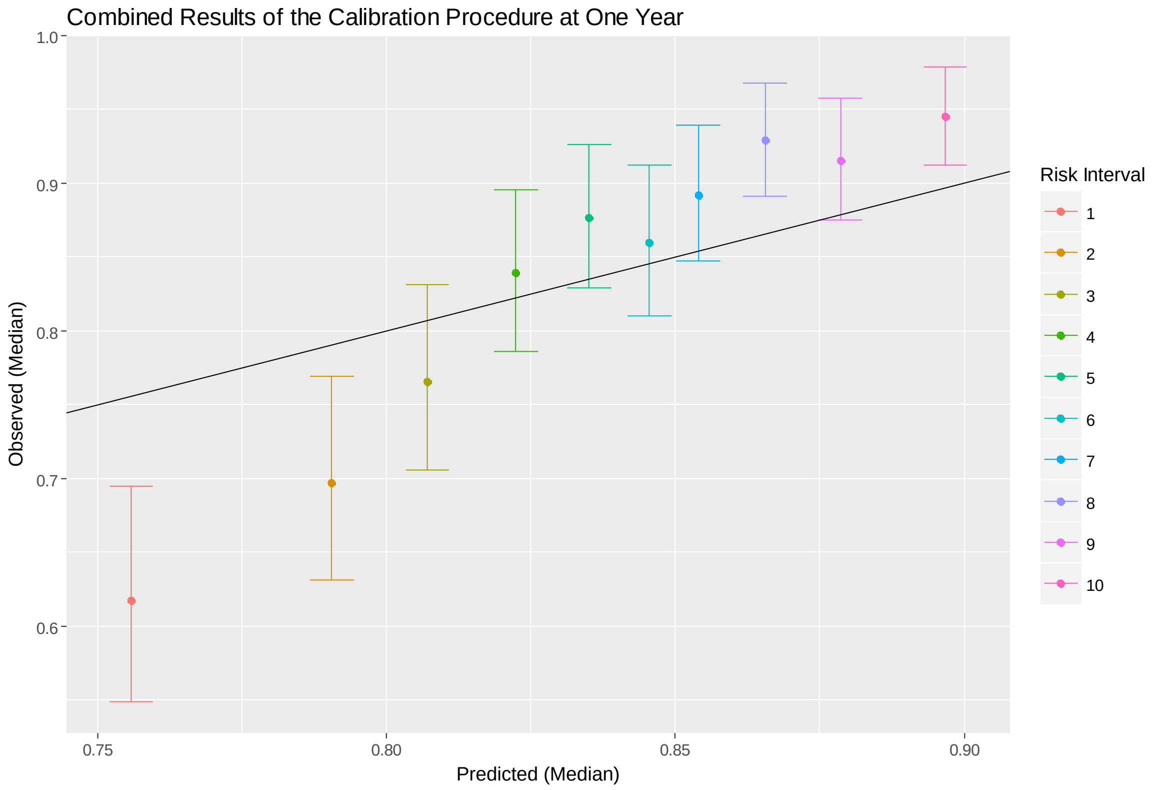

Supplementary Figure 2: MRP Scoring Algorithm

1. WHO PS
   a. Obtain the score associated with the participants WHO Performance Status from the look up table.
2. Age
   a. Obtain the patients age (in years) at baseline.
   b. Calculate $\frac{Age-74\cdot4}{5\cdot40} \times 0 \cdot 089 =$ _______
3. ISS
   a. Obtain the score associated with the participants ISS stage from the look up table.
4. CRP
   a. Obtain the patients CRP level (in mg/L) at baseline.
   b. Calculate $\frac{\log_e(CRP+1)-2\cdot08}{1\cdot11} \times 0 \cdot 035 =$ _______
5. Overall Score
   a. Calculate 1a + 2b + 3a + 4b = _______
   b. Categorise the individual into "Fit" "Intermediate-Fitness" or "Frail" using the score obtained in 5a and the MRP categorisation table.

**WHO Performance Status Look Up Table**	
WHO Performance Status	Score
0	-0.398
1	-0.199
2	0.000
3	0.199
4	0.397

**ISS Look Up Table**	
Stage	Score
I	-0.212
II	0
III	0.212

**MRP Categorization Table**	
Category	Cut-off
Fit	5a < -0·256
Intermediate-Fitness	-0·256 ≤ 5a ≤ -0·0283
Frail	5a > -0·0283

Supplementary Figure 3: The distributions of the MRP groups for the remaining Quality of Life subscales of the EORTC QLQ-C30 and QLQ-MY24 in MRC-IX at baseline.

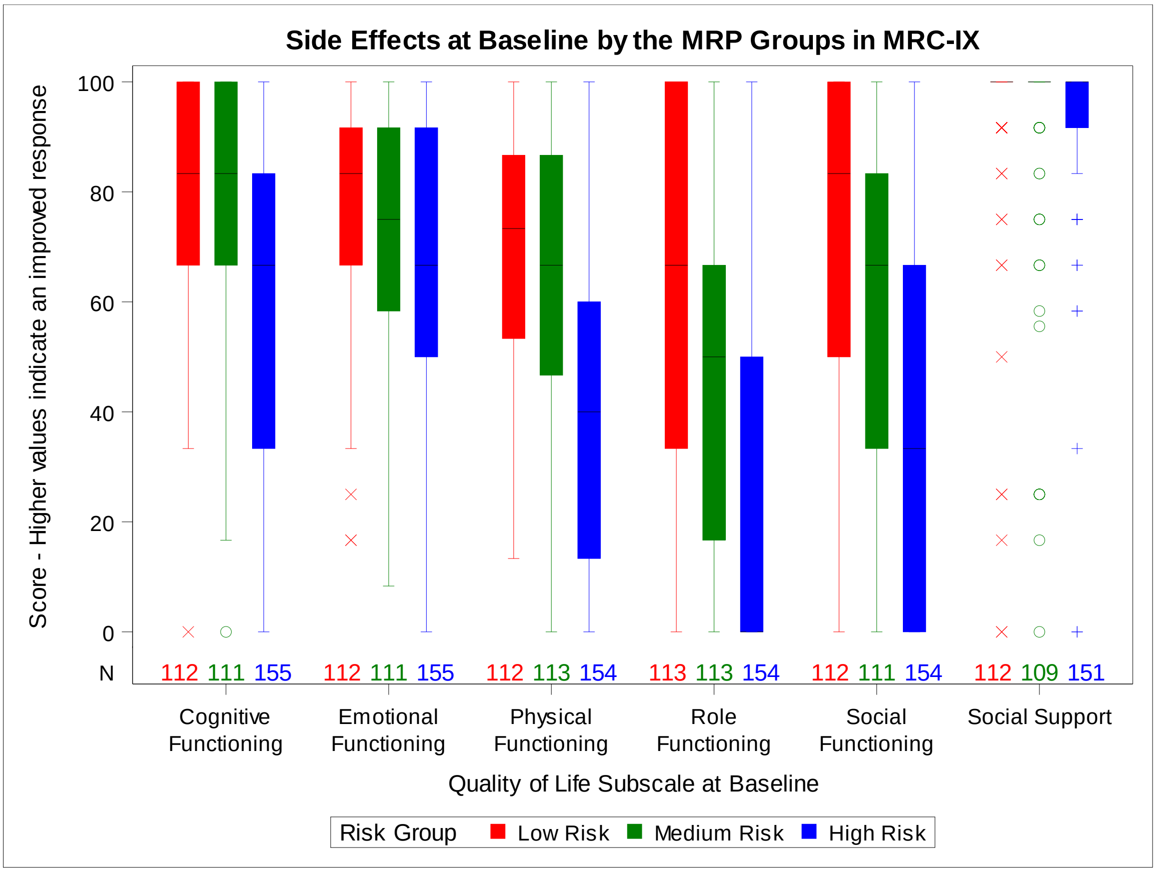

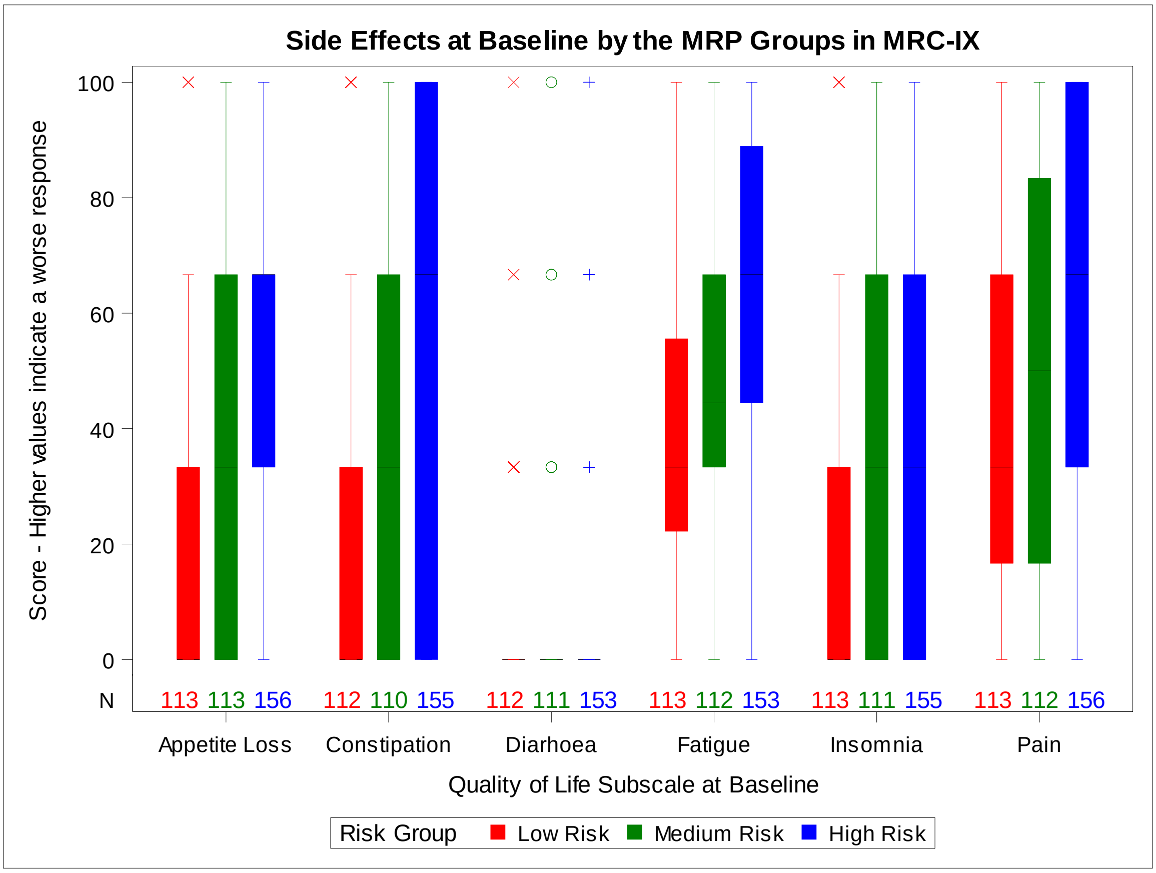

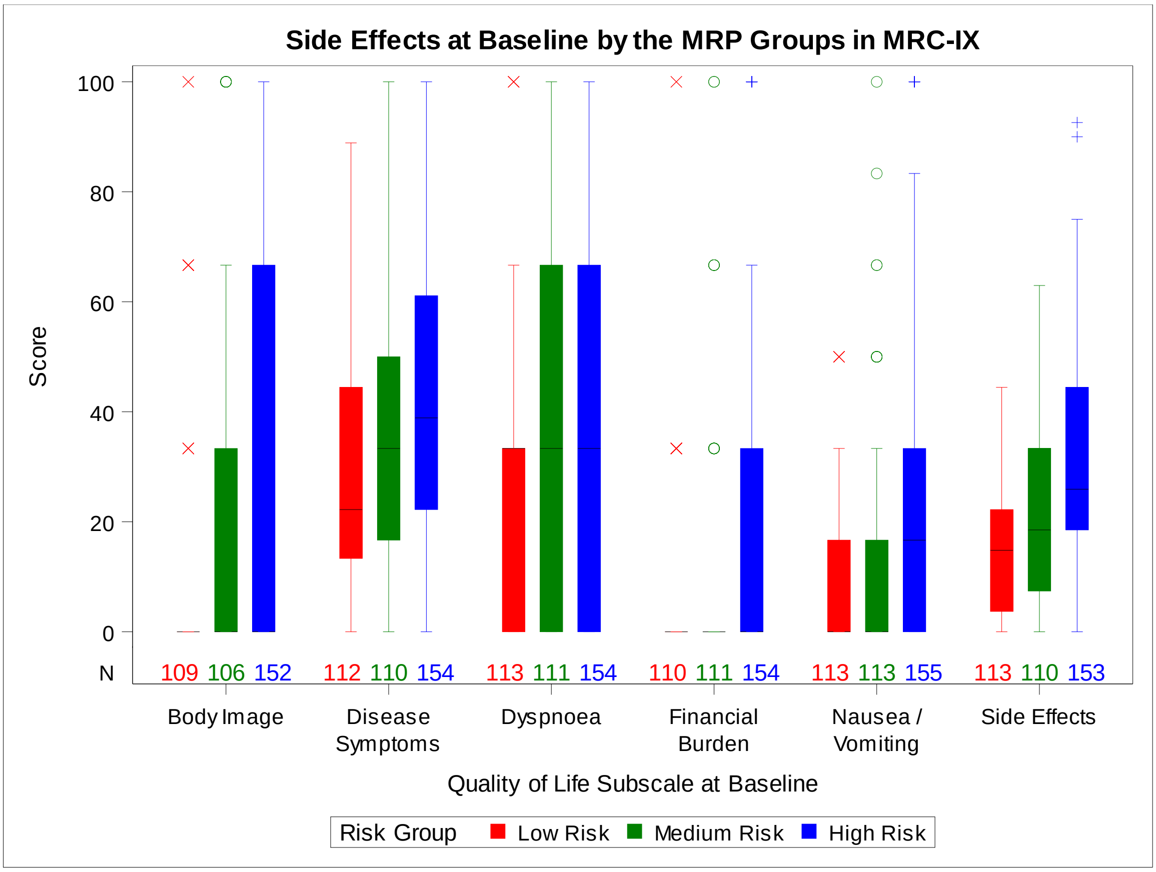
Side Effects at Baseline by the MRP Groups in MRC-IX
Score
100
80
60
40
20
0
N
109 106 152
112 110 154
113 111 154
110 111 154
113 113 155
113 110 153
Body Image
Disease Symptoms
Dyspnoea
Financial Burden
Nausea / Vomiting
Side Effects
Quality of Life Subscale at Baseline
Risk Group
Low Risk
Medium Risk
High Risk

Supplementary Figure 4: Kaplan-Meier Curves for overall survival (OS) by the MRP groups in MRC-IX with A) Standard (n=169), B) High-Risk (n=120) cytogenetic profiles. Standard risk was defined as the absence of any of the risk lesions; t(4;14), t(14; 16), t(14;20), del(17p) and gain(1q), high-risk at least one risk lesion (UK definition).

A)

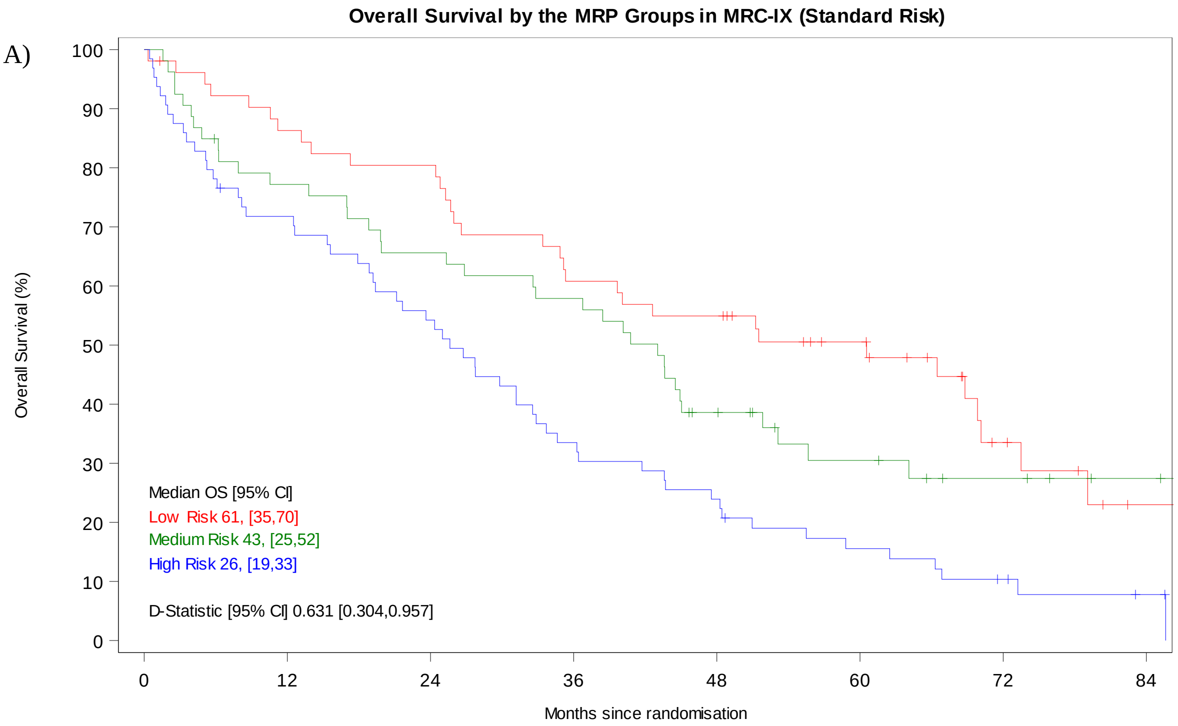

Number at risk (number censored)

	0	12	24	36	48	60	72	84
Low Risk	52 (0)	44 (1)	41 (1)	31 (1)	28 (1)	20 (7)	8 (14)	2 (18)
Medium Risk	53 (0)	40 (1)	34 (1)	30 (1)	18 (3)	11 (7)	7 (10)	4 (13)
High Risk	64 (0)	45 (1)	34 (1)	21 (1)	15 (1)	9 (2)	5 (3)	2 (5)

B)

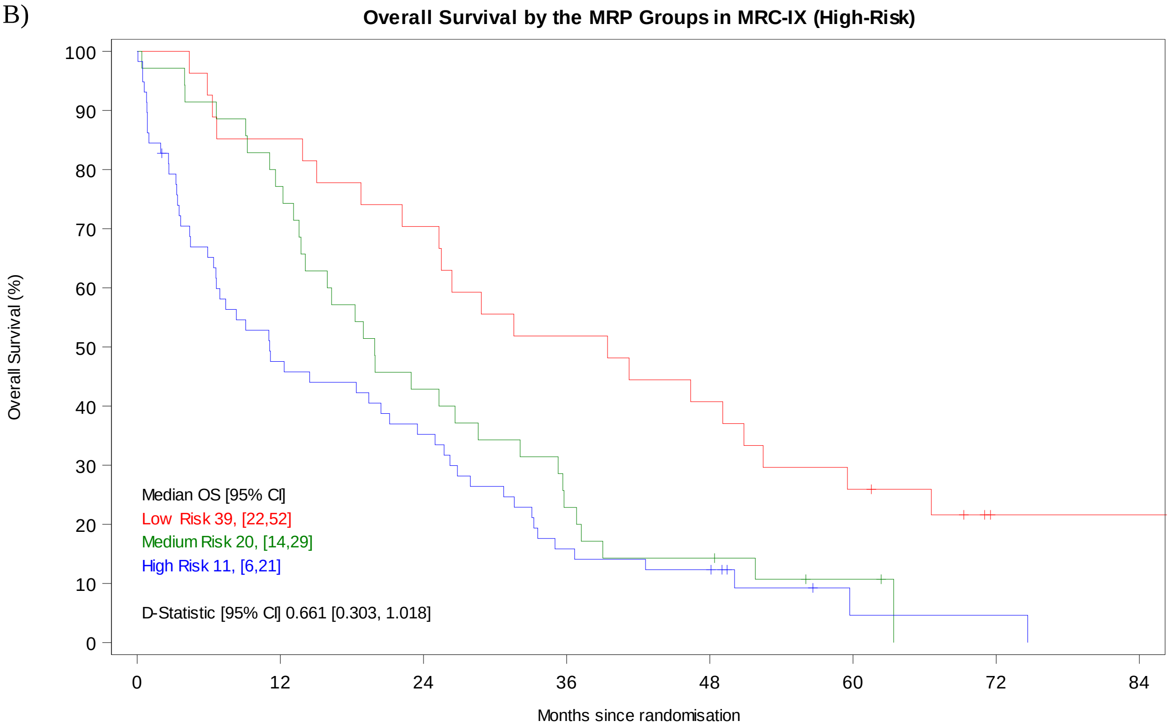

Number at risk (number censored)

	0	12	24	36	48	60	72	84
Low Risk	27 (0)	23 (0)	19 (0)	14 (0)	11 (0)	7 (0)	2 (4)	2 (4)
Medium Risk	35 (0)	27 (0)	15 (0)	8 (0)	5 (0)	2 (2)	0 (3)	
High Risk	58 (0)	27 (1)	20 (1)	9 (1)	7 (1)	1 (5)	1 (5)	0 (5)

Supplementary Figure 5: Kaplan-Meier curves for overall survival by the MRP groups for the patients in MRC-IX with A) Standard (n=231), B) High-Risk (n=58) cytogenetic profiles and patients in NCRI-XI with C) Standard (n=598) and D) High-Risk (n=133). Standard risk was defined as the absence of any of the risk lesions; t(4;14), t(14;16), and del(17p) high-risk at least one risk lesion (IMWG definition).

A)

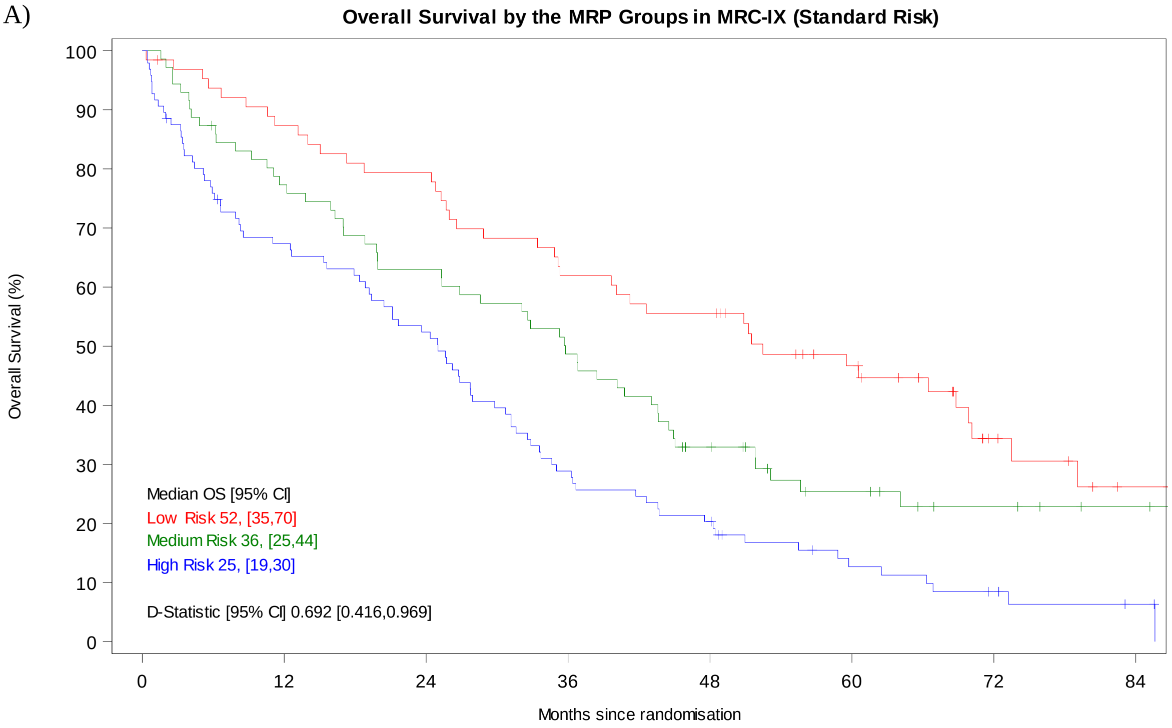

Number at risk (number censored)

	0	12	24	36	48	60	72	84
Low Risk	64 (0)	55 (1)	50 (1)	39 (1)	35 (1)	24 (7)	10 (16)	4 (20)
Medium Risk	71 (0)	54 (1)	44 (1)	34 (1)	21 (3)	12 (8)	7 (12)	4 (15)
High Risk	96 (0)	63 (2)	49 (2)	27 (2)	19 (2)	9 (6)	5 (7)	2 (9)

B)

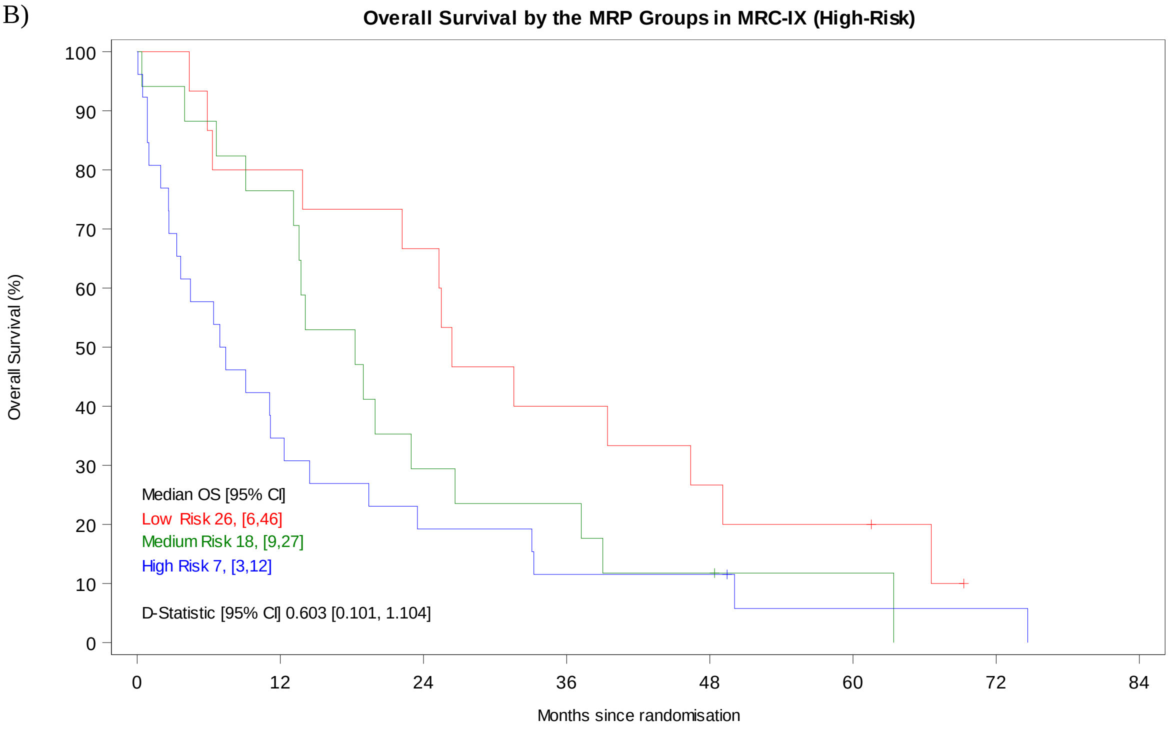

Number at risk (number censored)

	0	12	24	36	48	60	72	84
Low Risk	15 (0)	12 (0)	10 (0)	6 (0)	4 (0)	3 (0)	0 (2)	
Medium Risk	17 (0)	13 (0)	5 (0)	4 (0)	2 (0)	1 (1)	0 (1)	
High Risk	26 (0)	9 (0)	5 (0)	3 (0)	3 (0)	1 (1)	1 (1)	0 (1)

C) **Overall Survival by the MRP Groups in NCRI-XI (Standard Risk)**

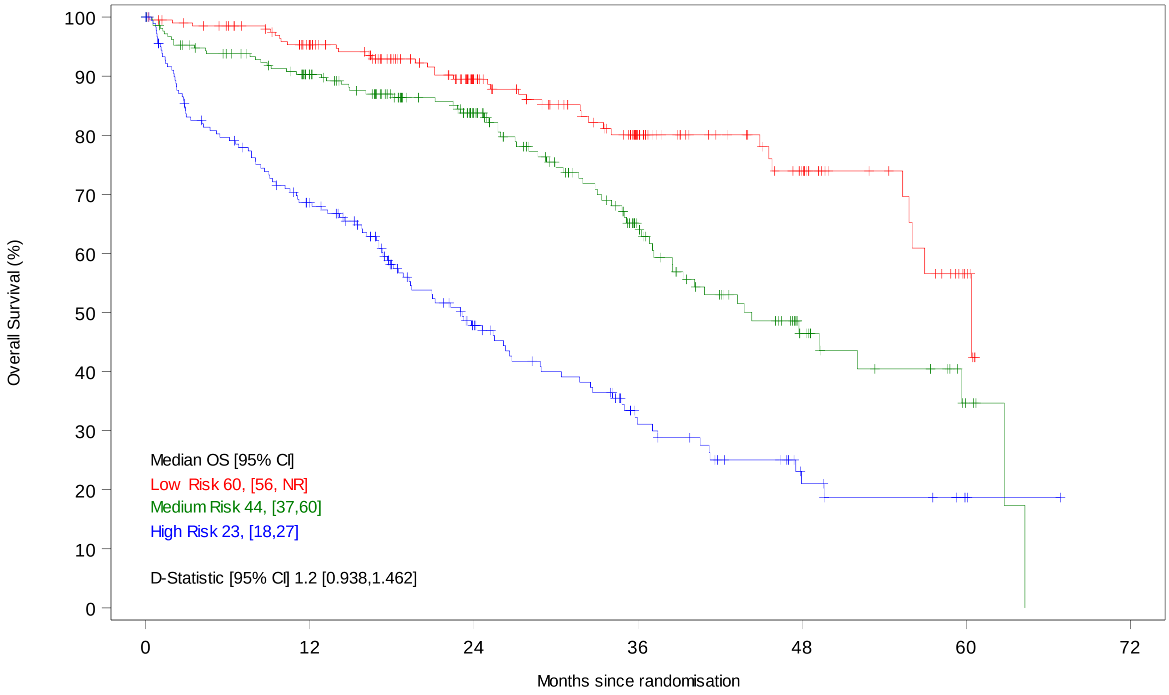

Number at risk (number censored)

	0	12	24	36	48	60	72
Low Risk	201 (0)	167 (25)	113 (70)	62 (111)	30 (140)	6 (160)	0 (165)
Medium Risk	215 (3)	169 (26)	114 (70)	58 (105)	20 (129)	4 (142)	0 (144)
High Risk	182 (1)	112 (15)	60 (37)	27 (52)	10 (62)	2 (69)	0 (71)

D) **Overall Survival by the MRP Groups in NCRI-XI (High-Risk)**

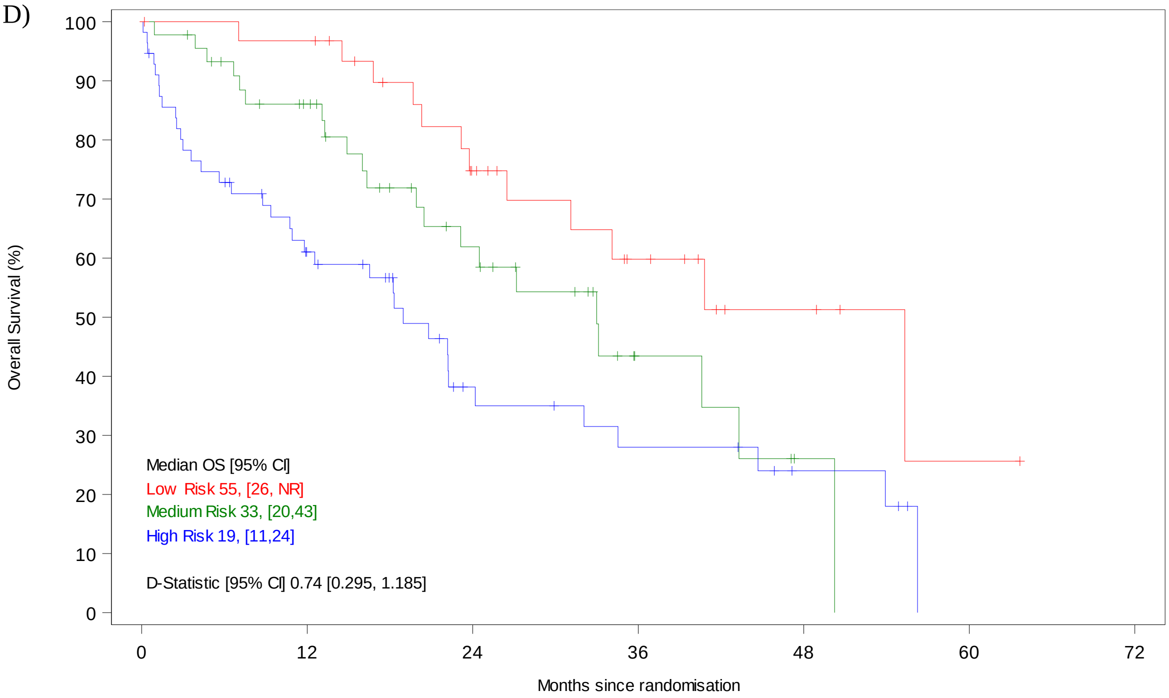

Number at risk (number censored)

	0	12	24	36	48	60	72
Low Risk	32 (0)	30 (1)	18 (7)	10 (12)	4 (17)	1 (19)	0 (20)
Medium Risk	45 (0)	33 (6)	18 (13)	5 (22)	1 (24)	0 (24)	
High Risk	56 (0)	29 (6)	12 (14)	8 (15)	4 (18)	0 (20)	

Supplementary Tables

Supplementary Table 1: Distribution of each potential prognostic factor across the MRP groups in the training (NCRI-XI) and test (MRC-IX) datasets.

		NCRI-XI: Training Dataset			MRC-IX: Test Dataset		
		**Low Risk (N=617)**	**Medium Risk (N=618)**	**High Risk (N=617)**	**Low Risk (N=142)**	**Medium Risk (N=150)**	**High Risk (N=228)**
L:W Ratio	Median (IQR)	0·294 (0·216,0·389)	0·286 (0·204,0·371)	0·233 (0·163,0·317)	0·296 (0·239, 0·371)	0·282 (0·207, 0·345)	0·245 (0·162, 0·328)
	Missing	0	4	3	0	1	1
Age	Median (IQR)	72 (69,76)	75 (71,77)	77 (73,80)	70 (67, 73)	73 (70, 76)	75 (72, 78)
CRP (mg/L)	Median (IQR)	4 (2,6·2)	5 (2·625,11·980)	12 (5,31)	5 (3,8·75)	7 (4·05, 11·82)	14·5 (5, 37·17)
	Missing	98	96	68	0	0	0
LDH (IU/L)	Median (IQR)	270 (182,317)	273 (191, 381·5)	296 (197,425·5)	316·5 (239·8, 409·5)	340 (253·0, 416·0)	329·50 (242·5, 434·2)
	Missing	128	138	150	46	45	68
WHO Performance Status N (%)	0	354 (57·4)	118 (19·1)	6 (1)	67 (47·2)	26 (17·3)	0
	1	218 (35·3)	365 (59·1)	227 (36·8)	68 (47·9)	102 (68)	68 (29·8)
	2	18 (2·9)	85 (13·8)	239 (38·7)	7 (4·9)	21 (14)	83 (36·4)
	3	0	4 (0·6)	106 (17·2)	0	1 (0·7)	67 (29·4)
	4	0	0	11 (1·8)	0	0	10 (4·4)
	Missing	27 (4·4)	46 (7·4)	28 (4·5)	0	0	0
ISS N (%)	I	269 (43·6)	39 (6·3)	10 (1·6)	54 (38·0)	10 (6·7)	4 (1·8)
	II	277 (44·9)	307 (49·7)	147 (23·8)	76 (53·5)	76 (50·7)	63 (27·6)
	III	36 (5·8)	209 (33·8)	414 (67·1)	12 (8·5)	64 (42·7)	161 (70·6)
	Missing	35 (5·7)	63 (10·2)	46 (7·5)	0	0	0

CRP: C-reactive protein, ISS: International Staging System, IQR: Interquartile range, L:W Ratio: ratio of lymphocytes to total white cells, LDH: Lactate dehydrogenase, .
Note that due to the inclusion criteria for the test dataset, only individuals with Age, CRP, WHO Performance Status and ISS recorded were included in the test dataset.

Supplementary Table 2: Logistic regression models for early mortality

	NCRI-XI: Training Dataset			MRC-IX: Test Dataset		
	**Estimate (SE)**	**Odds ratio (SE)**	**95% CI for OR**	**Estimate (SE)**	**Odds ratio (SE)**	**95% CI for OR**
Low Risk (Reference level)	-4·01 (0·30)			-4·25 (0·71)		
Medium Risk	0·76 (0·37)	2·14 (1·45)	(1·04, 4·42)	0·65 (0·87)	1·92 (2·39)	(0·35, 10·54)
High Risk	1·56 (0·34)	4·76 (1·40)	(2·44, 9·27)	2·36 (0·74)	10·59 (2·10)	(2·48, 45·17)

OR: odds ratio

References

1. Chiecchio L, Protheroe R, Ibrahim A, et al: Deletion of chromosome 13 detected by conventional cytogenetics is a critical prognostic factor in myeloma. Leukemia 20:1610, 2006
2. Boyle EM, Proszek PZ, Kaiser MF, et al: A molecular diagnostic approach able to detect the recurrent genetic prognostic factors typical of presenting myeloma. Genes, Chromosomes and Cancer 54:91-98, 2015
3. Kaiser M, Walker B, Hockley S, et al: A TC classification-based predictor for multiple myeloma using multiplexed real-time quantitative PCR. Leukemia 27:1754, 2013
